# The effect of frailty on hospitalisation trajectories in adults aged 65 and older across 10 European countries: a 14-year longitudinal analysis from the Survey of Health, Ageing, and Retirement in Europe (SHARE)

**DOI:** 10.1007/s10433-026-00914-z

**Published:** 2026-04-17

**Authors:** Selam Woldemariam, Erwin Stolz, K. Viktoria Stein, Sandra Haider, Thomas E. Dorner

**Affiliations:** 1 Karl Landsteiner Institute for Health Promotion Research, Kirchstetten, Austria; 2 Department for Social and Preventive Medicine, Center for Public Health, Medical University of Vienna, Vienna, Austria; 3 European Centre for Social Welfare Policy and Research, Vienna, Austria; 4 Institute of Social Medicine and Epidemiology, Medical University of Graz, Graz, Austria; 5 Academy for Ageing Research, “Haus Der Barmherzigkeit”, Vienna, Austria

**Keywords:** Frailty, Hospital use, Ageing population, Longitudinal studies, Europe

## Abstract

Evidence indicates an association between frailty and hospitalisation. However, hospital admissions occur repeatedly over time in old age, and most research has only focussed on single hospital admission. This study examines how frailty influences subsequent hospital use—*both* the risk of hospital admission and the duration of overnight stays—among community-dwelling adults aged 65 years and older across ten European countries over a 14-year period. Seven waves of data from the Survey of Health, Ageing and Retirement in Europe (SHARE) were used. Frailty was measured at baseline (wave 1) using the sex-specific SHARE Frailty Instrument, classifying participants as robust, prefrail, or frail. Hospital admission and length of stay during the past 12 months were measured at waves 2, 4, 5, 6, 7, and 8. Logistic mixed models was fitted to estimate the risk of hospital admission, and negative binomial mixed models to calculate the duration of hospital stays. Baseline frailty was associated with an increased risk of hospital admission over 14 years, yet among those who were hospitalised, frailty was not strongly related to longer stays. These findings suggest that frailty predicts whether older adults are admitted to hospital but is less informative regarding how long they remain once hospitalised. Routine frailty screening in primary care may help identify those at greatest risk and enable better-targeted community interventions to prevent avoidable hospitalisations and improve health outcomes for older people.

**Supplementary Information:**

The online version contains supplementary material available at 10.1007/s10433-026-00914-z.

## Introduction

The impact of ageing population on healthcare services utilisation depends on the relationship between longevity and health, a relationship that remains unclear in ageing research (Christensen et al 2009). Three theoretical frameworks developed in the 1980s offer competing explanations of how health trajectories evolve as life expectancy increases. The compression of morbidity hypothesis argues that advances in prevention and treatment delay the onset of disease, thereby reducing the period of ill health in later life (Fries 1980, 2003). In contrast, the expansion of morbidity hypothesis contend that longer life expectancy is accompanied by periods of illness, driven largely by the cumulative effects of unhealthy lifestyles and the increasing prevalence of chronic conditions across the life course (Gruenberg 1977). Positioned between these perspectives, the dynamic equilibrium hypothesis suggests that medical progress may simultaneously reduce disease severity and increase life expectancy without necessarily shortening disease duration (Manton 1982). Which means that the overall burden of ill health may remain relatively stable despite improvements in survival.

These competing theories impose different pressures on healthcare systems and long-term care provision. Thus, it is important for healthcare providers and policy makers to examine the long-term patterns of healthcare utilisation. The type and intensity of healthcare services used by older adults—such as hospital care or long-term care (LTC)—depend on whether additional life years are spent in good or poor health (Christensen et al. 2009). Current empirical evidence on health trends among older adults is mixed, partly due to differences in study design (such as cross sectional versus longitudinal data), study population coverage (the inclusion of institutionalised individuals), health outcome measures, data collection methods, and the time periods examined (Freedman et al. 2004; Parker and Thorslund 2007; Crimmins and Beltrán-Sánchez 2011).

Old age is often accompanied by multiple geriatric conditions, placing older adults at a high risk of hospitalisation, and a risk that increases further with advancing age (Launay et al 2018; Marcusson et al. 2020; Seaman et al. 2020). Hospital admission itself may exacerbate vulnerability in older adults, increasing the likelihood of adverse outcomes such as adverse drug reactions, hospital-acquired infections, delirium, falls, functional decline, rehospitalisation, disruption of care, institutionalisation, and mortality (van Vliet et al. 2017; Dent et al. 2023; Kojima 2016). Although hospitalisation is often necessary to treat acute illness and complex medical conditions, it is associated with rising healthcare costs (Bloom et al. 2015; Leonardi et al. 2014). This represents a growing public health concern, as older adults are the fastest-growing demographic group globally (Bloom et al. 2015; Leonardi et al. 2014). Given the resource-intensive nature of healthcare and the complex social and physical needs of frail older adults—who become increasingly reliant on family and professional caregivers—it is imperative for healthcare providers and policymakers to prevent the hospitalisation of older people.

Frailty has also been found as a predictor of hospitalisation among older adults (Avila-Funes et al. 2009; Dent et al. 2016; Roe et al. 2017; Woods et al. 2005), but also as a predictor of all-cause and cause-specific mortality (Grabovac et al. 2019; Wen et al., 2017). It is a clinical syndrome characterised by exhaustion, muscle weakness, unintended weight loss, slowness, and low physical activity, and is closely linked to biological ageing (Fried et al. 2001; Clegg et al. 2013; Rohrmann 2020). A recent meta-analysis of population-based studies from 62 countries estimated the prevalence of physical frailty at 12% among community-dwelling adults aged 50 years and older, although prevalence varied significantly across countries, geographic regions, and operationalisation of frailty (O’Caoimh et al. 2021). Despite multiple measurement of frailty, the frailty phenotype has consistently been associated with adverse health outcomes including hospitalisation (Fried et al. 2001; Avila-Funes et al. 2009; Dent et al. 2016; Roe et al. 2017; Woods et al. 2005). This association is further supported by systematic reviews and meta-analyses (Kojima 2016; Vermeiren et al. 2016). For instance, a meta-analysis of 31 longitudinal studies reported that frailty, regardless of the assessment instrument, significantly increased the likelihood of hospitalisation among community-dwelling adults aged 65 years and older (OR 1.8 95% CI [1.5–2.2]) among community-dwelling older adults aged 65 and above (Vermeiren et al. 2016).

In light of global ageing population, long-term outcome data are needed for healthcare systems to adequately respond to the needs of frail older adults. While previous studies have established that frailty is associated with an increased risk of hospital admission, most have focussed either on specific diagnoses (Penninx et al. 2006), length of stay from a single hospital admissions (Avila-Funes et al. 2009; Roe et al. 2017; Woods et al. 2005)**,** country-specific population (Roe et al. 2017)**.** Furthermore, studies often indicate just whether or not an individual was hospitalised, ignoring the duration of the stay (Roe et al. 2017) or have been limited to short follow-up period of three years (Avila-Funes et al. 2009)**.** Moreover, hospitalisation is often measured dichotomously (i.e. nights in hospital: yes/no), rather than as a continuous metric variable (Roe et al. 2017). As a result, it remains unclear whether frailty is associated not only with the risk of hospital admission but also with the accumulated burden of hospital use over time. By examining both—risk hospital admission and duration of overnight stays together—we provide a more comprehensive understanding of the sustained healthcare needs among older adults living with frailty and inform ongoing discussions on healthcare system capacity and resource planning in ageing populations across Europe.

The present study aims to analyse the relationship between frailty status and the risk of hospitalisation *and* duration of hospital days among community-dwelling older adults aged 65 and above in 10 European countries over a 14-year period.

## Methods

### Data and participants

The Survey of Health, Ageing, and Retirement in Europe (SHARE) is a large, on-going, cross-national longitudinal panel survey of community-dwelling individuals aged 50 years and older (Börsch-Supan et al. 2013). Since its initial wave in 2004/05, data on participants’ demographics, health, and socio-economic characteristics have been collected repeatedly every two years. To date, nine survey waves have been conducted. The present study used data from waves 1 through 8, excluding wave 3 due to the lack of information on hospitalisation data. Following the first wave in 2004–2005, subsequent interviews (waves) were conducted after 2 years in 2006–2007 (wave 2), 6 years in 2010–2012 (wave 4), 8 years in 2013 (wave 5), 10 years in 2015 (wave 6), 12 years in 2017/18 (wave 7), and 14 years in 2019/20 (wave 8). In this study, we focussed on older adults aged 65 and older, who are at higher risk for frailty. Participants were included in the analysis if data on frailty at baseline (wave 1) were available. Of the initial participants, 9.6% (1066) died within 14 years of follow-up after the first interview. The final sample consisted of 7249 participants, contributing a total of 22,127 observations across the seven waves in 10 European countries: Austria, Belgium, Denmark, France, Germany, Italy, The Netherlands, Spain, Sweden, and Switzerland.

### Frailty

Frailty was measured at baseline (wave 1) using the SHARE-Frailty-Instrument (SHARE-FI) (Romero-Ortuno et al. 2010), a sex-specific validated instrument for men and women, based on the five criteria of the Fried frailty phenotype: weight loss, exhaustion, weakness, slowness, and low physical activity (Fried et al. 2001). Weight loss was assessed through two questions. Participants were first asked, “What has your appetite been like?” with possible responses of “Diminution in desire for food” or “No diminution in desire for food.” For non-specific answers, they were asked, “So, have you been eating less than usual?” A response of “less” was coded as 1; otherwise, all other responses were coded as 0. Exhaustion was measured by a single-item question, “In the last month, have you had too little energy to do the things you wanted to do?” (yes = 1 and no = 0). Weakness was determined by grip strength using a Smedley dynamometer (TTM, Tokyo, 100 kg) (Wallis et al. 2015). Two consecutive measurements were taken for each hand, and the highest of the four values was recorded in kilograms (kg). Slowness was measured as a positive response to either of the following questions: “Because of health problems, do you have difficulty walking 100 m?” or “Climbing one flight of stairs without resting?” (yes = 1 and no = 0). Low physical activity was measured with the question, “How often do you engage in activities that require a low or moderate level of energy such as gardening, cleaning the car, or going for a walk?” Responses were categorised as: 1 = more than once a week; 2 = once a week; 3 = one to three times a month; 4 = hardly ever or never. Based on the five items, the SHARE-FI calculates a discrete factor that is used as a cutoff value to group participants in frailty categories separately for men and women.

### Hospitalisation

Two measures of hospitalisation—hospital admission and length of hospital stay—were selected from waves 2, 4, 5, 6, 7, and 8. At each wave, participants were asked: “During the last 12 months, have you been in a hospital overnight for medical, surgical, psychiatric reasons, or any other specialised wards?” (yes = 1 and no = 0). If participants answered “yes,” they were then asked: “How many nights altogether have you spent in hospitals during the last twelve months?”. Based on these responses, the total number of hospital days in the last year was calculated for each observation, recorded as a count variable ranging from 1 to 100.

### Covariates

Covariates included age (in years), sex (male/female), and marital status (coded as married or otherwise). Education was categorised into primary, secondary, and tertiary levels according to the International Standard Classification of Education (ISCED-97) (UNESCO 1997). Self-report multimorbidity was defined as the presence of two or more of the following chronic conditions: stroke, heart attack, hypertension, high blood cholesterol, diabetes mellitus, chronic lung disease, bronchial asthma, cancer, arthritis, osteoporosis, duodenal ulcer, Parkinson’s disease, cataracts, and hip fracture (coded has multimorbidity = 1 and otherwise = 0). All covariates were selected from study participants baseline (wave 1).

### Statistical analysis

Participants’ baseline characteristics were described by baseline frailty status and sex. Differences between the analysed and excluded samples were evaluated using chi-square tests or t tests, as appropriate. These comparisons reflect cumulative patterns of attrition over the 14-year follow-up period.

The distribution of hospital days showed a high number of zero values, as only a minority of participants reported any hospital days. The outcome variable, hospital days, was discrete, non-negative, and highly right-skewed, i.e. only a minority reported any hospital days, which is a common characteristic in healthcare utilisation studies (Layte and Nolan 2015; Smith et al. 2014). To address over-dispersed nature of the outcome variable, we used a two-part modelling strategy. First, we used logistic mixed-effect regression to model whether older adults reported any hospital admission (yes/no). Second, for those who were hospitalised, we applied a negative binomial model was used to assess the number of hospital days (Payne et al. 2018). Results are presented as Odds Ratios (OR) and Rate Ratios (RR), respectively. In addition, we calculated predicted probabilities of hospital admission and predicted number of hospital days for figure illustration (Table 1).

**Table 1:** Wave 1 characteristics of SHARE participants aged ≥ 65 years by frailty category and sex

Overall total	Men (			Women		
	*N* = 3353			*N* = 3896		
	Robust	Prefrail	Frail	Robust	Prefrail	Frail
	*n* = 2960 (88.3%)	*n* = 333 (9.9%)	*n* = 60 (1.8%)	n = 2424 (62.2%)	*n* = 948 (24.3%)	*n* = 524 (13.5%)
Age, mean (SD)	72.3 (5.8)	74.8 (6.6)	74.1 (5.79)	71.9 (5.6)	74.70 (6.3)	77.2 (6.9)
*Marital status*						
Married	2431(82.1%)	249 (74.8%)	37 (61.7%)	1379 (56.9%)	460 (48.5%)	210 (40.1%)
Not Married	529 (17.9%)	84 (25.2%)	23 (38.3%)	1045 (43.1%)	488 (51.5%)	314 (59.9%)
*Education level*						
Primary	1579 (53.3%)	211 (63.4%)	42 (70.0%)	1515 (62.5%)	711 (75.1%)	451 (86.1%)
Secondary	806 (27.2%)	75 (22.5%)	15 (25.0%)	587 (24.2%)	168 (17.7%)	52 (9.9%)
Tertiary	575 (19.5%)	47 (14.1%)	3 (5.0%)	321(13.3%)	68 (7.2%)	21 (4.0%)
*Multimorbidity*						
**Yes**	1354 (45.8%)	225 (67.57%)	44 (73.3%)	1185 (48.9%)	629 (66.4%)	408 (78.1%)
No	1604 (54.2%)	108 (32.43%)	16 (26.7%)	1238 (51.1%)	319 (33.6%)	115 (21.9%)

Values are n (%) unless otherwise noted. Continuous variables: mean (SD)

Analyses were performed separately for men and women to estimate the probability and length of hospitalisation from wave 2 (2006/07) through wave 8 (2019/20), covering 14-year follow-up period, based on baseline frailty status, adjusted for age, and country differences. Country-level variation in hospital use was controlled for by including a dummy variable for each country. A random intercept accounted for dependencies between repeated observations within individuals. Potential cofounders, including marital status, education, and multimorbidity, were tested but not included in the final model, as they did not improve model fit. The final model adjusted for age and country differences, summarised in Table 2 consists of a two-part approach. A logistic regression model estimating the odds of having at least one hospital admission among individuals with no hospitalisations (zero days). A negative binomial model estimating the average number of hospital days among those who had been hospitalised (non-zero days). Finally, we also conducted supplementary analyses to examine age differences in associations by stratifying participants into age groups (65–69 [reference], 70–74, 75–79, 80–84, and ≥ 85) and including interaction terms between categorical age group and baseline frailty status. Results are presented in online supplemental Table S5.

**Table 2:** Results of logistic and negative binomial mixed models over 14-year follow-up period by sex

	Men		Women	
	Zeros (Logit [OR (95% CI])	Count (Logit [RR 95% CI])	Zeros (Logit [OR 95% CI])	Count (Logit [RR 95% CI])
*Fixed effect*				
Intercept	− 3.9*** [0.0 (0.0–0.0]	1.29 [3.65 (1.78–7.47)]	− 4.1*** [0.0 (0.0–0.0]	0.4 [1.5 (0.8–2.7)]
Robust	Ref	Ref	Ref	Ref
Prefrail	0.7*** [1.9 (1.6–2.5)]	0.1*** [1.1 (0.9–1.3)]	0.3*** [1.4 (1.2–1.6]	0.1* [1.1 (0.9–1.2)]
Frail	0.9*** [2.4 (1.4–3.9)]	0.1 [1.1 (0.7–1.6)]	0.4*** [1.5 (1.3–1.8)]	0.2*** [1.3 (1.1–1.5)]
Age	0.0*** [1.0 (1.0–1.1)]	0.0 [1.0 (1.0–1.0)]	0.0*** [1.0 (1.0–1.0)]	0.0*** [1.0 (1.0–1.0)]
Year	0.0*** [1.0 (1.0–1.1)]	0.0 [1.0 (0.9–1.1)]	0.0*** [1.0 (1.0–1.1)]	0.0 [1.0 (1.0–1.0)]
*Random effects*				
Intercepts	0.7	0.3	0.5	0.4
*Model fit*				
Log likelihood	− 4895.9	− 7123.1	− 5993.6	− 8373.2
Observations	9778	2061	12,147	2489
Participants	3338	1453	3875	1725

Logit coefficients (columns 1 & 3) were exponentiated to obtain Odds Ratios (OR), estimating the probability of hospital admissions among participants without hospitalisations (zeros days). For the count part, negative binomial coefficients (columns 2 & 4) were exponentiated to obtain Rate Ratios (RR), representing the estimated average number of hospital days in the past 12 months among hospitalised participants. Logit coefficient significance *p < 0.05, **p < 0.01, ***p < 0.001

Model fit was evaluated using log-likelihood, Akaike Information Criterion (AIC), and Bayesian Information Criterion (BIC). Statistical analyses were performed using the `lme4` and `glmmTMB` package in R software (version 4.3.6) (Brooks et al. 2024).

## Results

The final sample included 3,353 men and 3,896 women, with participants' baseline characteristics presented in Table 1. Among men, 88.3% were classified as robust, 9.9% as prefrail, and 1.8% as frail. In comparison, 62.2% of women were robust, 24.3% were prefrail and 13.5% were frail. Frail individuals in both sexes were more likely to have lower educational attainment and multimorbidity compared to prefrail and robust individuals. Compared with the analytic sample and participants who were excluded were generally older and had a higher burden of multimorbidity across both sexes (see Supplementary Table S1 and S2). This pattern is consistent with the natural attrition observed in aging cohorts.

Out of the initial respondents, 43.3% (1453 men) and 44.3% (1725 women) were hospitalised during the 14-year follow-up period. Frail hospitalised individuals generally reported higher average and median number of hospital days compared with prefrail and robust individuals in both sexes, although this pattern was not consistent in all waves among women (see Supplementary Tables S3 and S4). Initially, frail hospitalised individuals of both sexes reported higher median hospital days in wave 2 (2006/2007): 10 days [IQR 2–15] for men and 10 days [IQR 5–15] for women. Prefrail individuals had a similar but slightly lower median stay—8 days [IQR 5–17] for men and 7 days [IQR 3–12] for women—while robust individuals reported the shortest median stays of 6 days [IQR 3–14] for men and 6 days [IQR 3–12] for women. By wave 8 (2019/20), median hospital days had declined across all frailty groups, showing similar trends in both sexes: frail (5 days [IQR 3–6] in men; 6 days [IQR 2–14] in women), prefrail (3 days [IQR 3–8] in men; 4 days [IQR 2–12] in women), and robust (6 days [IQR 2–12] in men; 6 days [IQR 2–10] in women).

**Fig. 1:**
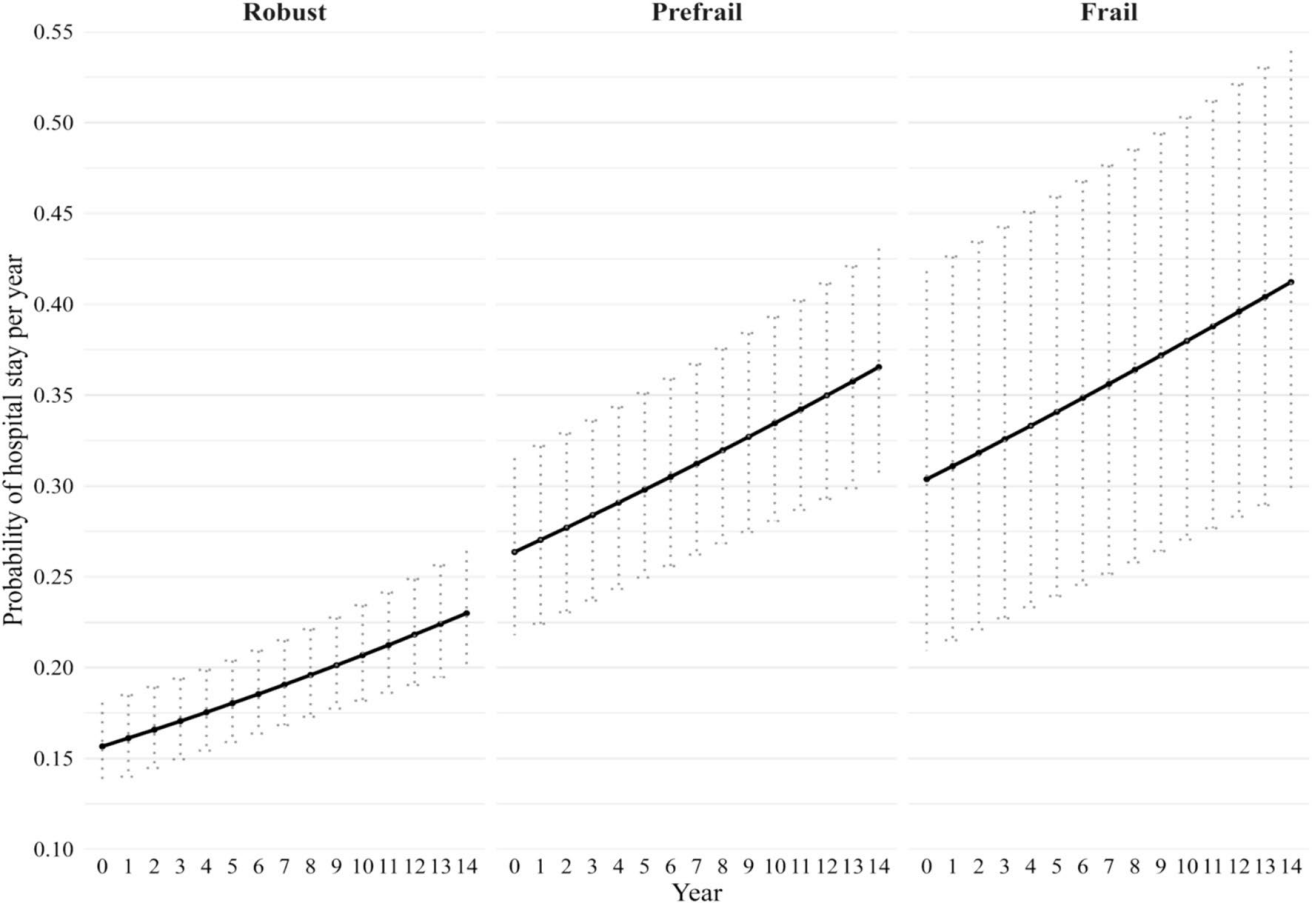
Estimated probability of hospitalisation among men over 14 years by frailty status, adjusted for age and country differences. Probabilities are based on the logit mixed model, with error bars representing the 95% CI

**Fig. 2:**
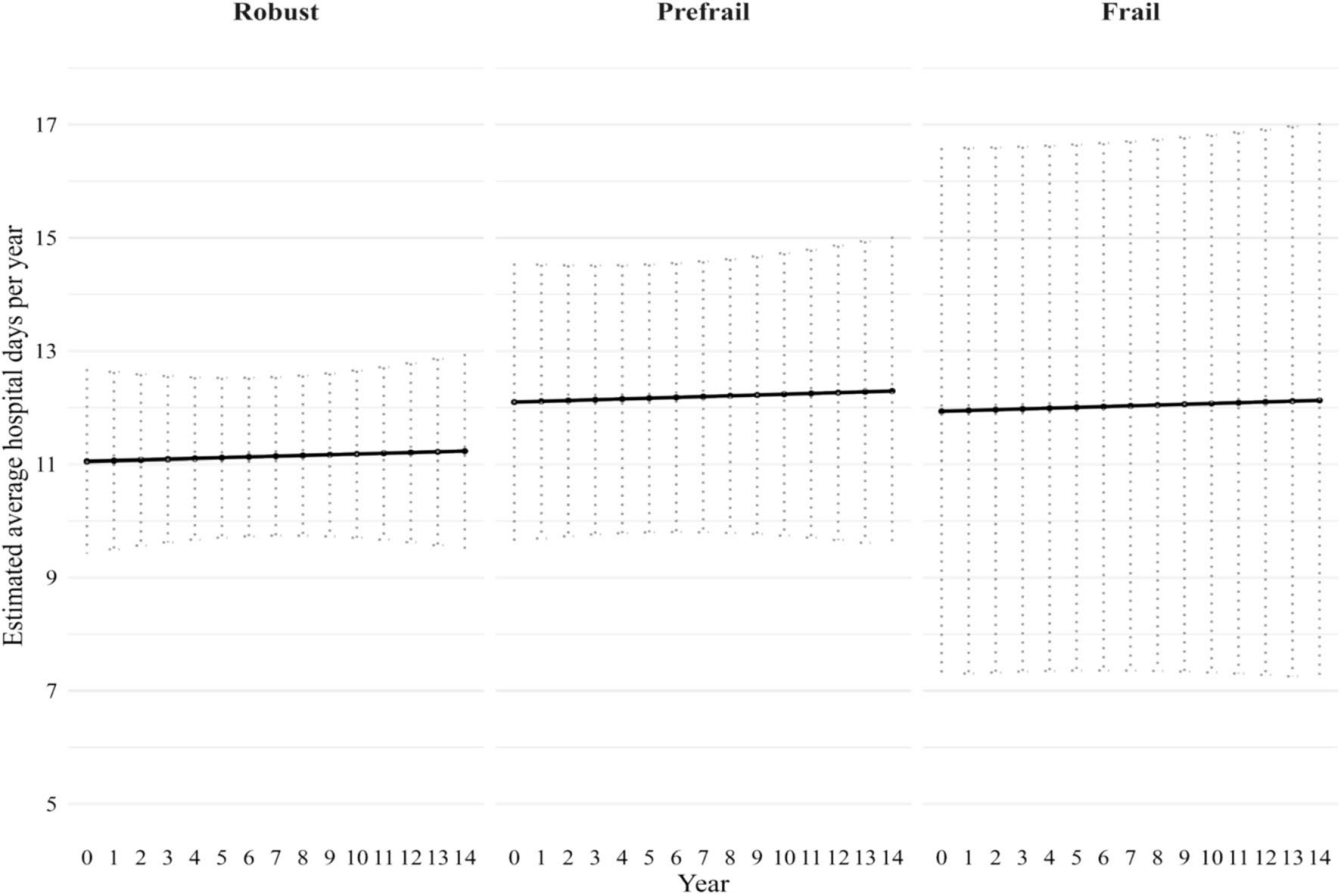
Estimated average length of hospital days among 1,453 hospitalised men over 14 years by frailty status, adjusted for age and country differences. Estimated number of hospital days are based on negative binomial mixed model. Error bars represent the 95% CI

Overall, frailty was associated with a higher likelihood of hospital admission, with steeper increases observed among frail individuals. Among men, frail participants had higher odds of hospital admission compared with robust men (OR 2.4; 95% CI 1.4–3.9), and prefrail men also demonstrated an elevated risk (OR 1.9; 95% CI 1.6–2.4).The probability of hospital admission among men by baseline frailty status over the 14-year period are shown in Fig. 1. Frail individuals consistently showed the highest risk of hospitalisation, starting at 32.4% (95% CI 22.0–41.8%) at baseline (year 0) and rising to 43.1% (95% CI 30.5–54.6%) by year 14. Similarly, prefrail men also experienced an increase in risk from 27.2% (95% CI 22.6–32.4%) to 37.1% (95% CI 31.1–43.6%) over the same period. Robust men had the lowest probability of hospitalisation, with a rate of 22.7% (95% CI 19.7–25.0%) by year 14. These patterns suggest that risk of hospitalisation increases with both frailty and advancing age, although frail individuals showed the widest confidence intervals, indicating greater variability (Fig. 2).

**Fig. 3:**
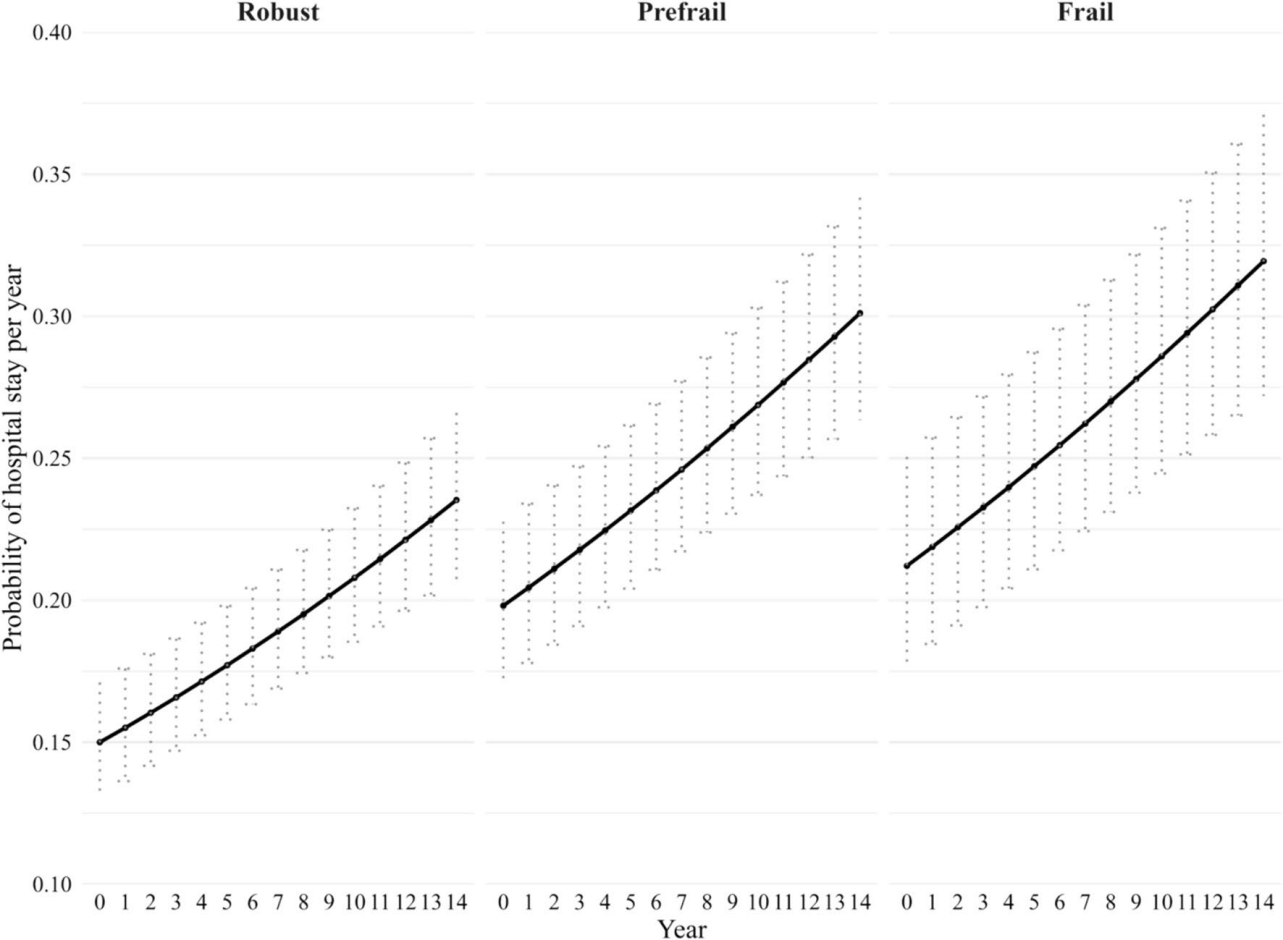
Estimated probability of hospitalisation among women over 14 years by frailty status, adjusted for age and country differences. Probabilities are based on the logit model, with error bars representing the 95% CI

Similar to the pattern observed in men, baseline frailty among women was associated with an increased risk of hospital admission over time. Frail women had higher odds of hospital admission compared with robust women OR 1.5; 95% CI 1.3–1.8), and prefrail women also showed an elevated risk (OR 1.4; 95% CI 1.2–1.6). As shown in Fig. 3, frail women had the highest and increasing probability of hospitalisation—22.3% (95% CI 18.7–26.4%) at baseline, linearly increasing linearly to 33.4% (95% CI 28.4–37.8%) by year 14. Prefrail women experienced a similar upward trend, from 19.8% (95% CI 17.2–22.6%) to 30.0% (95% CI 26.4–33.0%). With robust women showing the lowest likelihood of admission, with a rate of 23.2% (95% CI 20.5–26.1%) by year 14.Overall, these findings highlight frailty as a predictor of hospital admission, although the risk of hospital admission was lower among women than men. The positive associations between frailty and hospital admission remained consistent when using the categorical age bands and their interaction with frailty in models for both men and women (See supplementary Table S5).

**Fig. 4:**
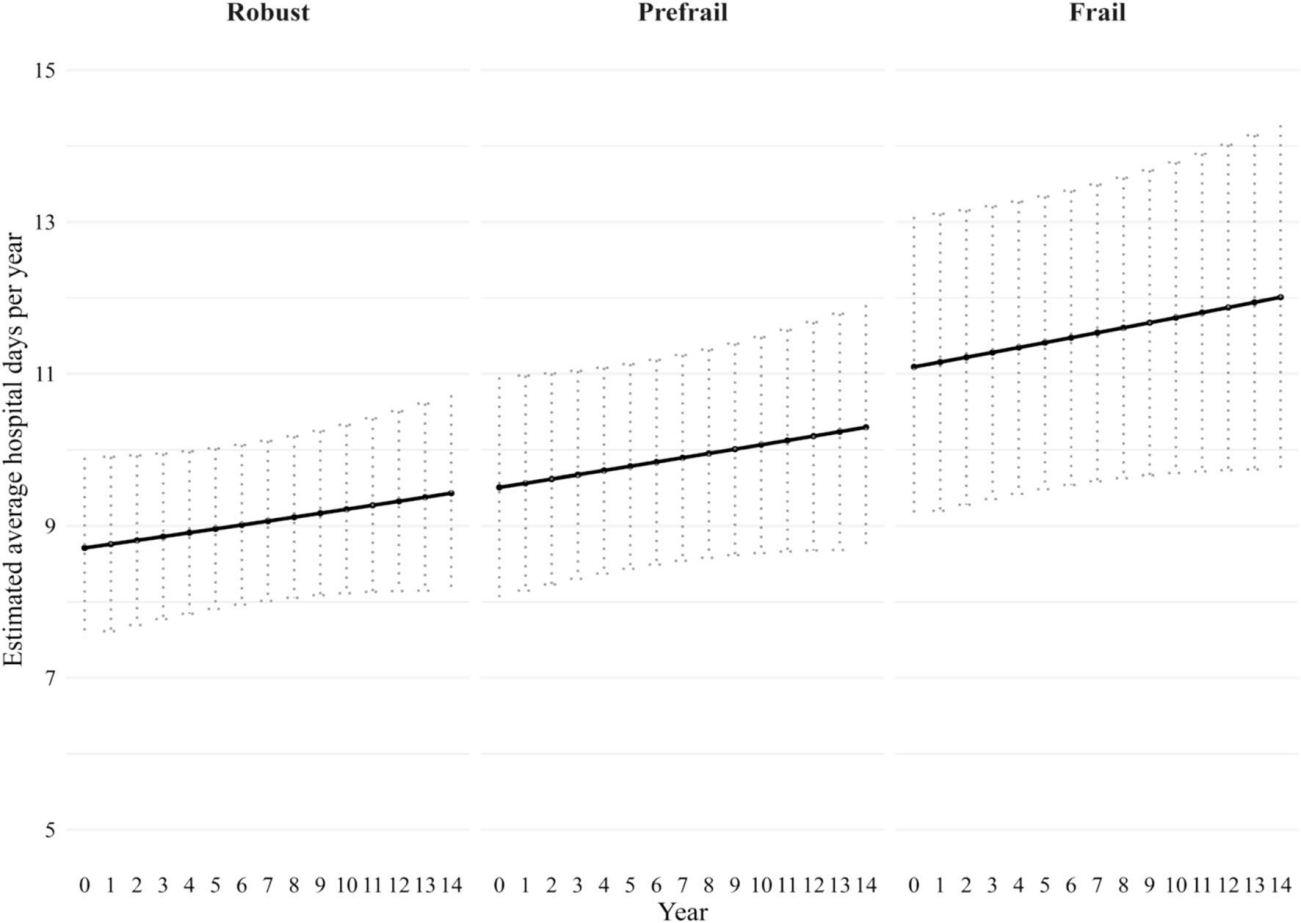
Estimated average length of hospital days among 1,725 hospitalised women over 14 years by frailty status, adjusted for age and country differences. Estimated number of hospital days are based on negative binomial mixed model. Error bars represent the 95% CI

However, we found that baseline frailty was associated with a slower rate of length of hospital stay over time. Across the 14-year follow-up, average hospital stay remained relatively stable across all frailty groups. As shown in Fig. 2, among the 1,453 hospitalised men, prefrail individuals had the longest average stay at year 14 (12.3 days; 95% CI 9.6–15.0), followed closely by frail individuals (12.1 days; 95% CI 7.2–17.0). Robust men had the relative shortest stays, averaging 11.2 days (95% CI 9.5–12.0) at year 14, corresponding to a difference of 1.1 days between frail and robust men. As shown in Fig. 4, among the 1,725 hospitalised women, as expected, frail participants had the longest hospital stays. Their average length of hospital stays stabilising around 11.1 days (95% CI 9.1–13.1) at baseline to 12.0 days (95% CI 9.7–14.3) at year 14. By year 14, frail women stayed an average of 2.6 days longer than robust women (12.0 vs 9.4 days; 95% CI 9.7–14.3 vs 8.2–10.7). Nevertheless, frailty had a less pronounced effect on length of hospital stays among hospitalised patients in both sexes.

## Discussion

The present study examined the effects of baseline frailty on the risk of hospital admission, and the duration of overnight hospital stays over a 14-year period in community-dwelling adults aged 65 years and older across 10 European countries. We found that for both men and women, frailty was associated with increased risk of hospitalisation and that the probability of hospital admission increased with advancing age during follow-up. In contrast, no significant differences were observed in the average length of hospital stays between frailty groups in both sexes. Together, these findings indicate that while frailty predicts the likelihood of being hospitalised, it has a comparatively smaller effect on length of hospital stay once admitted.

Results from the logistic model indicated frailty as a risk factor for hospital admission over time among community-dwelling older adults over time. Our findings align with previous research showing that frail and prefrail individuals face a higher risk of hospitalisation compared with their robust counterparts (Avila-Funes et al. 2009; Roe et al. 2017; Vermeiren et al. 2016; Woods et al. 2005). For instance, a systematic review found that the annual prevalence of hospitalisation among adults aged 65 and older ranges from 5.6 to 25.8% (Rodrigues et al. 2022). In our study, frail older adults had a higher probability of hospitalisation over 14-year follow-up period than robust individuals and experienced higher cumulative hospitalisation rate by year 14 (men: 43:1% [95% CI 30.5–54.6%] and women: 33.4% [95% CI 28.4–37.8%]).This could be the results of older adults, who are inherently more susceptible to adverse health outcomes, and frailty further exacerbates this vulnerability by reducing physiological reserve and resilience to stressors such as acute illness or injury (Dent et al. 2023; Hoogendijk et al. 2019; Kim and Rockwood 2024).

Results from the count models indicated that, among hospitalised individuals, differences in the duration of overnight hospital stays across frailty groups were relatively modest. This is broadly consistent with previous research showing that although frail individuals tend to report slightly longer hospital stays than robust individuals, the average differences are often small (Boucher et al. 2023; Hubbard et al. 2009; Munir Ehrlington et al. 2024; Wallis et al. 2015). For example, a prospective Swedish study reported slightly longer mean hospital stays among frail adults aged 65 years and older admitted through emergency departments compared with robust individuals (4.8 vs. 2.7 days) (Munir Ehrlington et al. 2024). Similarly, a UK study using the Fried frailty phenotype found minimal differences in total hospital days over a two-year period between frail and non-frail groups (Keeble et al. 2019). One possible explanation for the moderate differences observed in our study is that hospitalised individuals are already highly vulnerable and at elevated risk of mortality, which may reduce the total time spent in hospital. This suggests a potential ceiling effect in the predictive capacity of frailty measures when applied to particularly frail hospitalised populations and should be considered when interpreting associations with length of stay.

The findings of the present study have important implications for both clinical practice and public health. Hospitalisation among older adults itself, have been associated with an increased risk of subsequent functional decline, mobility and reduced quality of life (Goodwin et al. 2011; Graf 2006; van Vliet et al. 2017). These adverse outcomes can create a vicious cycle in which functional deterioration increases the likelihood of rehospitalisation and mortality, thereby further heightening vulnerability (Dent et al. 2023; Kojima 2016). Post-discharge functional decline and mobility limitations—often requiring rehabilitation or long-term care—not only compromise the health and independence of older individuals but also place additional strain on healthcare systems (Cygańska et al. 2023; Umegaki 2024).

Previous studies have emphasised the need for timely frailty screening interventions to improve quality of life in later years and thereby reducing avoidable hospitalisations (Dent et al. 2023; Haider et al. 2020; Luger et al. 2016). Although the frail population is heterogenous regarding care needs, incorporating clinical assessments of patients to identify high-risk individuals and improve patient outcomes is imperative. These implications may become more pronounced as the population of older adults grows and frailty becomes more prevalent among community-dwelling older adults.

The main limitation of this study is the sparse and unbalanced data, that is, the limited number of repeated observations and of reported hospital stays over the 14-year period. Frail participants—those at the highest risk of hospitalisation—may have died during the observation period, reducing the total number of observations in this subgroup. To address this, we included a variable (died during follow-up yes/no) between waves in the model estimation. Additionally, due to the longitudinal design in the study we included only respondents that had participated in two or more survey waves, which further may have exacerbated the non-response bias. Attrition due to dropout, a common limitation in longitudinal studies may have further influenced hospitalisation patterns, possibly underestimating the observed hospitalisation trajectories. Hospital admission data were self-reported, which may be influenced by recall bias, particularly among older adults. This limitation could result in misreporting of hospital use and should be considered when interpreting the findings. Lastly, the exclusion of participants with missing data on variables for measuring frailty, 11.8% (2316), from the original sample from Wave 1 might have biased an underestimate.

There is a lack of population-based empirical studies focussed on hospital service use among older adults over a long follow-up period (14 years). To our knowledge, this is the first study to examine both the risk of hospital admission and total length of hospital stay in the past 12 months over an extended period at the population level across Europe. By utilising data from SHARE—a comprehensive longitudinal study covering 10 European countries—we were able to analyse hospitalisation patterns and the total number of hospital days in a large sample of community-dwelling older adults 65 years and older. The use of repeated observation across several countries enhances the generalisability of our findings. This extensive dataset allowed us to identify long-term hospitalisation trends, such as higher hospitalisation risk among frail individuals.

## Conclusion

The present study examined the relationship between frailty status and the probability and duration of hospitalisation among community-dwelling adults aged 65 and older across 10 European countries over a 14-year period. Our findings demonstrate that frailty is a risk factor for hospitalisation. Over time, the risk of hospitalisation increased with advancing age, regardless of frailty status. Among those hospitalised, although both frail and prefrail hospitalised individuals had slightly longer hospitalisation compared with robust counterparts, there was small differences in hospital duration across frailty status. These findings point towards the importance of early identification and screening of prefrail and frail older adults in the community as a potential strategy to reduce avoidable hospital admissions, improve quality of life among older adults and alleviate healthcare system demands.

## Authors' contributions

SW, ES and TD conceived the original idea and the study design. SW and ES analysed the data and wrote the manuscript. ES, SH, VK, TD critically provided feedback on several drafts of this manuscript.

## Supplementary Information

Below is the link to the electronic supplementary material.

Supplementary file1 (DOCX 32 kb)

## Data Availability

No datasets were generated or analysed during the current study.
