# Supplementary material for: The effect of frailty on hospitalisation trajectories in adults aged 65 and older across 10 European countries: a 14-year longitudinal analysis from the Survey of Health, Ageing, and Retirement in Europe (SHARE)
Source: Eur J Ageing. 2026 Apr 17;23(1):25. doi: 10.1007/s10433-026-00914-z (PMC13216402; doi:10.1007/s10433-026-00914-z)
Supplement: Supplementary file 1 — Supplementary file1 (DOCX 32 kb) [file 10433_2026_914_MOESM1_ESM.docx]

**Supplementary Material**

**The effect of frailty on hospitalisation trajectories in adults aged 65 and older across 10 European countries: A 14-year longitudinal analysis from the Survey of Health, Ageing, and Retirement in Europe (SHARE)**

**Authors:** Selam Woldemariam, Erwin Stolz, Viktoria K. Stein, Sandra Haider, Thomas E. Dorner

**Table S1:** **Comparing covariates between the analysed sample and excluded sample among men.**

|  | **Analysed sample** | **Excluded sample*** | **P-value**  **(X^2^ or t-test)** |
| --- | --- | --- | --- |
| **Total** | N= 3,353 | N=4,993 |  |
| **Age, mean (SD)** | 72.6 ± 5.9 | 73.3 ± 6.3 | <0.001 |
| **Marital Status** |  |  | 0.323 |
| Married | 2,717 (81.1%) | 2,741 (56.0%) |  |
| Not Married | 636 (18.9%) | 2,241 (44.0%) |  |
| **Education level** |  |  | 0.412 |
| Primary | 1,832 (54.6%) | 2,741 (56.0%) |  |
| Secondary | 896 (26.7%) | 1,367 (27.4%) |  |
| Tertiary | 625 (18.7%) | 874 (16.6%) |  |
| **Multimorbidity** |  |  | 0.144 |
| **Yes** | 1,623 (48.4%) | 2,489 (50.1%) |  |
| No | 1,730 (51.6%) | 2,480 (49.9%) |  |

Data are presented as mean (SD) for continuous variables and n (%) for categorical variables.

*Participants aged < 65-years, those with missing data on SHARE-frailty instrument (FI) calculation at baseline (wave 1), and those lost to follow-up during 2006/7-2019/20 were excluded.

P-values are based on t-tests or χ² tests, as appropriate.

**Table S2:** **Comparing covariates between the analysed sample and excluded sample among women.**

|  | **Analysed sample** | **Excluded sample*** | **P-value**  **(X^2^ or t-test)** |
| --- | --- | --- | --- |
| **Total** | N= 3,896 | N=6,090 |  |
| **Age, mean (SD)** | 73.3 ± 6.3 | 74.2 ± 6.9 | <0.001 |
| **Marital Status** |  |  |  |
| Married | 2,049 (52.6%) | 3,037 (50.0%) | 0.012 |
| Not Married | 1847 (47.4%) | 3,040 (50.0%) |  |
| **Education level** |  |  | 0.243 |
| Primary | 1,832 (54.6%) | 4,247 (69.9%) |  |
| Secondary | 896 (26.7%) | 1,245 (20.5%) |  |
| Tertiary | 625 (18.7%) | 580 (9.6%) |  |
| **Multimorbidity** |  |  | 0.620 |
| **Yes** | 1,623 (48.4%) | 3,488 (57.6%) |  |
| No | 1,730 (51.6%) | 2,569 (42.4%) |  |

Data are presented as mean (SD) for continuous variables and n (%) for categorical variables.

*Participants aged < 65-years, those with missing data on SHARE-frailty instrument (FI) calculation at baseline (wave 1), and those lost to follow-up during 2006/7-2019/20 were excluded.

P-values are based on t-tests or χ² tests, as appropriate.

**Table S3:** Distribution of hospital days in the past year by wave and frailty group among men.

| **Wave**  **(interview year)** | **Total**  **observation** | **Frailty**  **groups** | **Zero-count*** | **Non-zero**** | **Mean (SD)** | **Median (IQR)** |
| --- | --- | --- | --- | --- | --- | --- |
| 2 (2006/07) | 2662 | Robust | 2183 (82.0%) | 479 (18.0%) | 11.92 (14.9) | 6 (3-14) |
|  | 314 | Prefrail | 211 (67.2%) | 103 (32.8%) | 12.39 (10.1) | 8 (5-17) |
|  | 53 | Frail | 29 (54.7%) | 24 (45.3%) | 18.42 (29.1) | 10 (2-15) |
| 4 (2011/12) | 1907 | Robust | 1498 (78.6%) | 409 (21.4%) | 11.67 (13.8) | 7 (3-15) |
|  | 146 | Prefrail | 102 (69.9%) | 44 (30.1%) | 10.89 (11.6) | 8 (4-14) |
|  | 21 | Frail | 16 (76.2%) | 5 (23.8%) | 23.17 (24.8) | 14 (8-15) |
| 5 (2013) | 1627 | Robust | 1321 (81.2%) | 306 (18.8%) | 12.92 (16.1) | 7 (3-15) |
|  | 115 | Prefrail | 83 (72.2%) | 32 (27.8%) | 16.25 (20.5) | 8 (4-15) |
|  | 14 | Frail | 11 (78.6%) | 3 (21.4%) | 8.67 (5.0) | 8 (6-11) |
| 6 (2015) | 1221 | Robust | 955 (78.2%) | 266 (21.8%) | 10.64 (12.0) | 6 (3-14) |
|  | 75 | Prefrail | 48 (64.0%) | 27 (36.0%) | 16.22 (15.7) | 10 (7-20) |
|  | 11 | Frail | 10 (90.9%) | 1 (9.1%) | 22.00 (--) | 22 (--) |
| 7 (2017) | 957 | Robust | 749 (78.3%) | 208 (21.7%) | 11.65 (14.4) | 7 (3-15) |
|  | 42 | Prefrail | 32 (76.2%) | 10 (23.8%) | 12.00 (12.5) | 8 (3-14) |
|  | 7 | Frail | 4 (57.1%) | 3 (42.9%) | 1.67 (0.6) | 2 (1.50-2) |
| 8 (2019/2020) | 576 | Robust | 444 (77.1%) | 132 (22.9%) | 9.93 (12.3) | 6 (2-12) |
|  | 27 | Prefrail | 20 (74.1%) | 7 (25.9%) | 5.57 (5.2) | 3 (3-8) |
|  | 3 | Frail | 1 (33.3%) | 2 (66.7%) | 5.50 (0.7) | 5.5 (--) |

Values are n (%) unless otherwise noted. Continuous variables: mean (SD).

Mean and median days is calculated for hospitalised patients.

*Zero (n, %): Represent the number and percentage of individuals without hospitalisation in the past year.

**Non-Zero (n, %): Represent the number and percentage of hospitalised individuals in the past year.

**Table S4:** Distribution of hospital days in the past year by wave and frailty group among women.

| **Wave**  **(interview year)** | **Total observation** | **Frailty**  **groups** | **Zero-count***  **(No visit)** | **Non-zero**** | **Mean** | **Median (IQR)** |
| --- | --- | --- | --- | --- | --- | --- |
| 2 (2006/07) | 2164 | Robust | 1829 (84.5%) | 335 (15.5%) | 9.94 (11.6) | 6 (3-12) |
|  | 851 | Pre-frail | 661 (77.7%) | 190 (22.3%) | 10.91 (13.5) | 7 (3-12) |
|  | 458 | Frail | 337 (73.6%) | 121 (26.4%) | 14.64 (17.1) | 10 (5-15) |
| 4 (2011/12) | 1629 | Robust | 1330 (81.7%) | 299 (18.3%) | 10.48 (13.8) | 6 (3-12) |
|  | 616 | Pre-frail | 481 (78.1%) | 135 (21.9%) | 12.56 (14.9) | 7 (3-15) |
|  | 273 | Frail | 210 (76.9%) | 63 (23.1%) | 13.78 (16.0) | 8 (2-20) |
| 5 (2013) | 1463 | Robust | 1176 (80.4%) | 287 (19.6%) | 9.21 (11.4) | 6 (3-10) |
|  | 524 | Pre-frail | 395 (75.4%) | 129 (24.6%) | 13.04 (17.7) | 7 (3-15) |
|  | 220 | Frail | 168 (76.4%) | 52 (23.6%) | 12.33 (16.1) | 7 (3-13) |
| 6 (2015) | 1168 | Robust | 921 (78.9%) | 247 (21.1%) | 10.21 (12.2) | 7 (3-12) |
|  | 405 | Pre-frail | 308 (76.1%) | 97 (23.9%) | 11.07 (15.0) | 7 (3-14) |
|  | 159 | Frail | 126 (79.2%) | 33 (20.8%) | 17.03 (19.3) | 9 (3-20) |
| 7 (2017) | 946 | Robust | 741 (78.3%) | 205 (21.7%) | 11.04 (14.5) | 7 (3-14) |
|  | 307 | Pre-frail | 235 (76.6%) | 72 (23.4%) | 12.36 (15.0) | 7 (3-14) |
|  | 110 | Frail | 85 (77.3%) | 25 (22.7%) | 17.20 (19.3) | 10 (7-20) |
| 8 (2019/2020) | 615 | Robust | 480 (78.1%) | 135 (21.9%) | 8.44 (9.5) | 6 (2-10) |
|  | 174 | Pre-frail | 127 (72.9%) | 47 (27.1%) | 12.62 (20.2) | 4 (2-12) |
|  | 65 | Frail | 48 (73.9%) | 17 (26.1%) | 12.06 (17.5) | 6 (2-14) |

Values are n (%) unless otherwise noted. Continuous variables: mean (SD).

Mean and median days is calculated for hospitalised patients.

*Zero (n, %): Represent the number and percentage of individuals without hospitalisation in the past year.

**Non-Zero (n, %): Represent the number and percentage of hospitalised individuals in the past year.

**Table S5.** Logistic and negative binomial mixed models examining the association between baseline frailty and risk of hospital admission, and length of hospital stay, including categorical age bands and their interaction with frailty groups, in men and women.

|  | **Men** | | **Women** | |
| --- | --- | --- | --- | --- |
|  | Zeros  (Logit [OR (95% CI]) | Count  (Logit [RR 95% CI]) | Zeros  (Logit [OR 95% CI]) | Count  (Logit [RR 95% CI]) |
| Fixed effect |  |  |  |  |
| Intercept | -1.9*** [0.2 (0.1−0.2] | 2.3*** [10.0 (8.5−11.8)] | -1.9*** [0.1 (0.1−0.2] | 1.9*** [7.1 (6.1−8.2)] |
| Robust | REF | REF | REF | REF |
| Prefrail | 0.9*** [2.7 (1.9−3.9)] | -0.3*** [0.9 (0.7−1.3)] | 0.4*** [1.5 (1.2−1.9)] | 0.2* [1.3 (1.0−1.6)] |
| Frail | 0.98 [2.7 (0.9−7.4)] | -0.2 [0.8 (0.4−1.7)] | 0.5** [1.7 (1.2−2.4)] | 0.5** [1.7 (1.2−2.3)] |
| Age (65-69) | REF | REF | REF | REF |
| 70-74 | 0.2** [1. (1.1−1.5)] | 0.3 [1.0 (0.9−1.2)] | 0.2* [1.2 (1.1−1.5)] | 0.2* [1.2 (1.0−1.4)] |
| 75-79 | 0.5*** [1.6 (1.3−1.9)] | 0.1 [1.1 (0.9−1.3)] | 0.5*** [1.6 (1.3−1.9)] | 0.3*** [1.4 (1.2−1.6)] |
| 80-84 | 0.5*** [1.6 (1.3−2.1)] | 0.3** [1.4 (1.1−1.7)] | 0.7*** [2.1 (1.6−2.7)] | 0.5*** [1.6 (1.3−1.9)] |
| 85+ | 0.6** [1.8 (1.2−2.9)] | 0.0 [1.0 (0.7−1.5)] | 0.8*** [2.3 (1.4−3.6)] | 0.9*** [2.5 (1.8−3.7)] |
| Year | 0.0*** [1.0(1.0−1.1)] | 0.0 [1.0 (0.9−1.0)] | 0.0*** [1.04 (1.0−1.1)] | 0.0 [1.0 (0.9−1.0)] |
| Prefrail * age (70-74) | -0.6* [0.67 (0.3−0.9)] | 0.3 [1.4 (0.9−2.1)] | -0.1 [0.9 (0.6−1.2)] | -0.2 [0.8 (0.6−1.1] |
| Prefrail * age (75-79) | -0.5 [0.6 (0.3−1.1)] | 0.1 [1.2 (0.7−1.8)] | -0.0 [0.9 (0.7−1.4)] | -0.2 [0.8 (0.6−1.1)] |
| Prefrail * age (80-84) | -0.4 [0.9 (0.5−1.9)] | -0.1 [0.9 (0.5−1.5)] | -0.4 [0.7 (0.4−1.0)] | -0.1 [0.9 (0.6−1.3)] |
| Prefrail * age (85+) | -1.1 [0.3 (0.1−1.1)] | 0.6 [1.9 (0.7−4.8)] | -0.5 [0.6 (0.3−1.2)] | -0.7 [0.5 (0.3−0.9)] |
| Frail * age (70-74) | -0.3 [0.8 (0.2−2.8)] | 0.4 [1.6 (0.9−2.1)] | 0.1 [1.1 (0.6−1.2)] | -0.3 [0.8 (0.5−1.1)] |
| Frail * age (75-79) | 0.5 [1.6 (0.4−6.5)] | -0.0 [0.9(0.4−2.7)] | -0.1 [0.9 (0.6−1.5)] | -0.3 [0.8 (0.5−1.2)] |
| Frail * age (80-84) | -0.9 [0.4 (0.1−2.6)] | 1.7* [5.6 (1.3−14.3)] | -0.3 [0.8 (0.5−1.3)] | -0.4* [0.7 (0.5−1.1)] |
| Frail * age (85+) | -0.6 [0.5 (0.3−11.9)] | -1.4 [0.3 (0.0−3.4)] | -0.8* [0.4 (0.2−0.9)] | -0.9* [0.4 (0.2−0.8)] |
| Random effects |  |  |  |  |
| Intercepts | 0.7 | 0.3 | 0.5 | 0.4 |
| Model fit |  |  |  |  |
| AIC | 9830.0 | 14284.6 | 12023.7 | 16779.3 |
| BIC | 10016.9 | 14436.6 | 12216.3 | 16936.5 |
| Log likelihood | -4889.0 | -7123.1 | -5985.9 | -8362.7 |
| Observations | 9778 | 2061 | 12147 | 2489 |
| Participants | 3338 | 1453 | 3875 | 1725 |

Logit coefficients (columns 1 & 3) were exponentiated to obtain Odds Ratios (OR), estimating the probability of hospital admissions among participants without hospitalisations (zeros days). For the count part, negative binomial coefficients (columns 2 & 4) were exponentiated to obtain Rate Ratios (RR), representing the estimated average number of hospital days in the past 12 months among hospitalised participants. Logit coefficient significance *p<0.05, **p<0.01, ***p<0.001.
